# Combination Treatment of Tamoxifen with Risperidone in Breast Cancer

**DOI:** 10.1371/journal.pone.0098805

**Published:** 2014-06-02

**Authors:** Wei-Lan Yeh, Hui-Yi Lin, Hung-Ming Wu, Dar-Ren Chen

**Affiliations:** 1 Department of Cell and Tissue Engineering, Changhua Christian Hospital, Changhua, Taiwan; 2 School of Pharmacy, China Medical University, Taichung, Taiwan; 3 Department of Neurology, Changhua Christian Hospital, Changhua, Taiwan; 4 Comprehensive Breast Cancer Center, Changhua Christian Hospital, Changhua, Taiwan; China Medical University, Taiwan

## Abstract

Tamoxifen has long been used and still is the most commonly used endocrine therapy for treatment of both early and advanced estrogen receptor-positive breast cancer in pre- and post-menopause women. Tamoxifen exerts its cytotoxic effect primarily through cytostasis which is associated with the accumulation of cells in the G_0_/G_1_ phase of the cell cycle. Apoptotic activity can also be exerted by tamoxifen which involves cleavage of caspase 9, caspase 7, caspase 3, and poly-ADP-ribose polymerase (PARP). Down-regulation of anti-apoptotic proteins Bcl-2 and Bcl-x_L_ and up-regulation of pro-apoptotic proteins Bax and Bak have also been observed. In addition, stress response protein of GRP 94 and GRP 78 have also been induced by tamoxifen in our study. However, side effects occur during tamoxifen treatment in breast cancer patients. Researching into combination regimen of tamoxifen and drug(s) that relieves tamoxifen-induced hot flushes is important, because drug interactions may decrease tamoxifen efficacy. Risperidone has been shown to be effective in reducing or eliminating hot flushes on women with hormonal variations. In this present study, we demonstrated that combination of tamoxifen with risperidone did not interfered tamoxifen-induced cytotoxic effects in both *in vitro* and *in vivo* models, while fluoxetine abrogated the effects of tamoxifen. This is the first paper suggesting the possibility of combination treatment of tamoxifen with risperidone in breast cancer patients, providing a conceivable resolution of tamoxifen-induced side effects without interfering the efficacy of tamoxifen against breast cancer.

## Introduction

Breast cancer is one of the most common cancers among American women, and it also is the second leading cause of cancer death in women. Estimated by National Cancer Institute, about 1 in 8 women in the US will develop invasive breast cancer during their lifetime, and the chance that breast cancer will be responsible for a woman's death is 1 in 36 (http://seer.cancer.gov/csr/1975_2010/). Approximately 70% of breast cancers express estrogen receptor (ER) as ER-positive primary tumors, and most of these breast cancers depend on estrogen signaling for their growth and survival [1], [2]. Endocrine therapy aims to switch off estrogen signaling in ER-positive breast cancer cells to halt cell proliferation and induce cell death [3], [4], [5].

Tamoxifen (Tam) is a selective estrogen receptor modulator (SERM), it binds to ER as partial agonist or antagonist in a manner depend on target tissue [6], [7]. Tamoxifen has long been used and still is the most commonly used endocrine therapy for treatment of both early and advanced ER-positive breast cancer in pre- and post-menopause women [8], [9], [10], [11]. However, side effects are the unwanted effects of the treatment. Ongoing side effects, such as hot flushes and sweats, fatigue, painful joints, and mood changes not only can greatly decrease quality of life, but they may lead to discontinuation of the therapies [12], [13], [14]. Similar symptoms were relieved by selective serotonin reuptake inhibitors (SSRIs) in post-menopause women with hormonal variations, however, SSRIs has been reported to have negative drug interactions with tamoxifen due to disturbing tamoxifen metabolism. As a prodrug, tamoxifen is metabolized in the liver mainly by CYP2D6 isoenzyme to two active metabolites, 4-hydroxytamoxifen (4-OH-Tam) and 4-hydroxy-N-desmethyltamoxifen (endoxifen) [15]. Inhibition of CYP2D6 decreases tamoxifen metabolism and adversely affects the efficacy against breast cancer treatment [16], [17]. Evidence shows that co-administration of CYP2D6 inhibitor like fluoxetine or paroxetine (both are SSRIs) decreases the plasma concentration of tamoxifen metabolites due to inhibition of CYP2D6 enzyme activity [18], [19]. Tamoxifen exerts its cytotoxic effect primarily through cytostatic rather than cytocydal action. It has been reported that tamoxifen-induced growth inhibition is associated with the accumulation of cells in the G_0_/G_1_ phase of the cell cycle [20]. Moreover, cytostasis, induced by cell cycle arrest, is a condition that is poorly tolerated by any cell and must either be escaped or resolved by cellular death, hence the apoptotic activity of these primarily cytostatic agents [21]. It has been reported that tamoxifen-induced apoptosis involves cleavage of caspase 9, caspase 7, caspase 3, and poly-ADP-ribose polymerase (PARP) [5], [22], [23]. Anti-apoptotic protein Bcl-2 and pro-apoptotic protein Bax are also important effectors in the regulation of tamoxifen-induced cell death [5], [24].

Risperidone is an anti-psychotic medication that functions by interfering with the communication among nerves in the brain. Risperidone is mainly metabolized to 9-hydroxyrisperidone (paliperidone) by CYP2D6 also [25], [26]. Risperidone acts as a 5-HT_2A_ antagonist and can be used to quickly and effectively block the effects of 5-HT_2A_ agonists at a low dose [27], [28]. Risperidone is also a potent dopamine type 2 (D_2_) and α_2_ adrenergic receptor antagonist [26]. Thus, risperidone has been used in the treatment of psychotic disorders, for example, schizophrenia, with a standard dose around 6 mg/day [28], [29]. However, risperidone has been found to be effective in other off-labeled effects in the past few years. It has been reported that risperidone increases inflammatory parameters and restores anti-inflammatory pathways in a model of neuroinflammation [30]. Risperidone has also been found to attenuate inflammatory responses to ameliorate pancreatitis [31]. Importantly, risperidone has been shown to be effective in reducing or eliminating hot flushes and other symptoms associated with hormonal variations on women at a respectively low dose of 1–2 mg/day without apparent side effects [32]. Unlike other commonly used SSRIs for these symptoms, risperidone does not inhibit CYP2D6 enzyme activity which is essential for tamoxifen metabolism [25], [33].

Researching into combination regimen of tamoxifen and drug(s) that relieves tamoxifen-induced side effects is important, because drug interactions may decrease tamoxifen efficacy. Whether risperidone interferes tamoxifen is unknown. In this present paper, we found that risperidone did not affect human breast cancer cell line T47D cells' viability. In combination with risperidone, tamoxifen-induced growth inhibition and cell cycle arrest of T47D cells were not interfered by risperidone both *in vitro* and *in vivo*. Stress responses of endoplasmic reticulum and protein expressions of apoptotic pathways were also examined, and none of these were influenced by co-administration of risperidone. Nevertheless, fluoxetine abrogated tamoxifen-induced cytostatic and apoptotic effects significantly. This is the first paper suggesting the possibility of combination treatment of tamoxifen and risperidone in breast cancer patients, providing a conceivable resolution of tamoxifen-induced side effects without interfering the efficacy of tamoxifen against breast cancer.

## Materials and Methods

### Materials

Goat anti-CYP2D6, rabbit anti-c-Myc, anti-cyclin D1, anti-Rb, anti-p-Rb, anti-PARP-1, anti-Bak, anti-Bax, anti-caspase-3, anti-caspase-9, anti-GRP 94, anti-GRP 78, mouse anti-Bcl-2 and anti-Bcl-xL antibodies were purchased from Santa Cruz (Santa Cruz, CA). Mouse anti-caspase-7, anti-mouse, anti-rabbit, and anti-goat horseradish peroxidase (HRP)-linked antibodies were purchased from Cell Signaling (Danvers, MA). Mouse anti-β-actin antibody, risperidone, paliperidone, tamoxifen, 4-hydroxytamoxifen, endoxifen, fluoxetine, crystal violet, 3-(4,5-cimethylthiazol-2-yl)-2,5-diphenyl tetrazolium bromide (MTT), sulforhodamine B (SRB), dimethyl sulfoxide (DMSO), propidium iodide (PI) were purchased from Sigma-Aldrich (St. Louis, MO). RNase A was purchased from Amresco (Solon, OH).

### Cell cultures

Human breast cancer cell lines T47D, MCF-7, MDA-MB-231 and human lung cancer cell line A549 were purchased from American Type Culture Collection (Manassas, VA). T47D cells, MCF-7 cells, and A549 cells were maintained in RPMI medium supplemented with 10% fetal bovine serum (FBS), 100 IU/ml penicillin, and 100 mg/ml streptomycin (Invitrogen, Carlsbad, CA) at 37°C in a humidified incubator under 5% CO_2_ and 95% air. Confluent cultures were passaged by trypsinization.

### Crystal Violet (CV) staining

Cell viability was determined by staining with crystal violet according to our previous report [1]. After indicated period of drug treatment, cells were washed with PBS twice and then fixed with 12% formaldehyde. After 10 minutes incubation at room temperature, formaldehyde was aspirated and cells were air dried for 20 minutes, followed by staining with 1% crystal violet in 50% methanol for 5 minutes. Stained cells were washed with tap water and subjected to spectrophotometric quantitation (OD 540 nm) using Thermo Multiskan® Spectrum plate reader.

### MTT assay

Live cells was measured by using the 3-(4,5-cimethylthiazol-2-yl)-2,5-diphenyl tetrazolium bromide (MTT) assay according to our previous study [34], [35]. Culture medium was aspirated after indicated treatment, and cells were washed with PBS twice. MTT solution (0.5 mg/ml in PBS) was then added in each culture well and cells were incubated at 37°C. After 1 hour's incubation, MTT solution was removed and cells were lysed by DMSO. The absorbance was measured at 550 nm by Thermo Multiskan® Spectrum plate reader.

### Sulforhodamine B (SRB) assay

The SRB assay is based on the measurement of cellular protein content according to our previous study [36], [37]. Culture medium was aspirated after indicated treatment, and cells were fixed with 10% trichloroacetic acid for 10 minutes. 0.4% (w/v) SRB in 1% acetic acid was then added in each culture well and stained for 30 minutes. Unbound SRB was washed out by 1% acetic acid and SRB-bounded cells were dissolved by 10 mM Tris solution. The absorbance was measured at 515 nm by Thermo Multiskan® Spectrum plate reader.

### Flow cytometry

Cells were washed with PBS and detached by trypsin at 37°C. After fixing by 70% ethanol over night at −20°C, cells were centrifuged for 5 minutes and supernatant was discarded. Cells were then rinsed with PBS and incubated with 10 µg/ml propidium iodide (PI) in 0.1% Triton X-100 solution supplemented with 0.02 mg/ml RNase A in the dark. After 1 hour's incubation, cell cycle was analyzed by Cytomics™ FC500 flow cytometer using CXP software (Beckman Coulter) [38].

### Western blot analysis

After washing with ice-cold PBS, cells were lysed with radioimmunoprecipitation (RIPA) assay buffer on ice for 30 min. After centrifugation at 14,000 g for 20 minutes, the supernatant was used for Western blot or stored at −20°C. Protein concentration was measured by BCA assay kit (Pierce, Rockford, IL) with BSA as standard. Equal proteins were separated on SDS-polyacrylamide gels and transferred to polyvinylidene difluoride (PVDF) membranes (Millipore, Billerica, MA). The membranes were incubated for 2 hours with 7.5% dry skim milk in PBS-Tween 20 buffer to block nonspecific binding and then incubated with primary antibodies over night. After washing with PBS-Tween 20, the membranes were incubated with HRP-conjugated secondary antibodies for another 1 hour. The blots were visualized by enhanced chemiluminescence (ECL; Santa Cruz Biotechnology) using classic blue autoradiography film (MIDSCI, St. Louis, MO) [39].

### Tumor xenograft

All animals were purchased from National Laboratory Animal Center and kept in a climate-controlled animal room. Experiments were conducted in strict accordance with the recommendations in the guide approved by the Institutional Animal Care and Use Committee of Changhua Christian Hospital (Permit Number: CCH-AE-99-023). During experiments, all efforts were made to minimize animal suffering. Tumor xenografts were established by injection of T47D breast cancer cells into the abdominal mammary gland of 8-week-old female BALB/c athymic nude mice. 1×10^7^ T47D cells suspended in 0.1 ml culture medium were inoculated by 1 ml syringe with 23G 1 1/4 needle (BD302008) beside the lowest right nipple, and the needle was left around 5 seconds to minimize back flow or solution loss on Day zero of the study. After 3 weeks, palpable tumor masses had been observed, and regimens were started at Day 42 when all tumors reached the volume of 100 mm^3^. The individual or combination regimens of each drug (25 µg tamoxifen per mouse or/and 2.5 µg risperidone per mouse) dissolved or suspended in 100 µl PBS were injected intraperitoneally (i.p.) every 2 days by 1 ml syringe and 25G 5/8 needle (BD302104). Tumor volume (tumor volume  =  length×width^2^×0.5) was recorded once a week following the equation utilized by Kotoh et al and Ruddy and Majumdar in similar tumor xenograft studies [40], [41], [42]. All mice were sacrificed at Day 91 by CO_2_ euthanasia.

### Statistics

Values are expressed as mean ± S.E.M. of at least three experiments. Results were analyzed by student's *t*-test and significance was defined as *p*<0.05.

## Results

### Tamoxifen exerts cytotoxic effect while risperidone does not affect cell viability in T47D breast cancer cells

**Figure 1:**
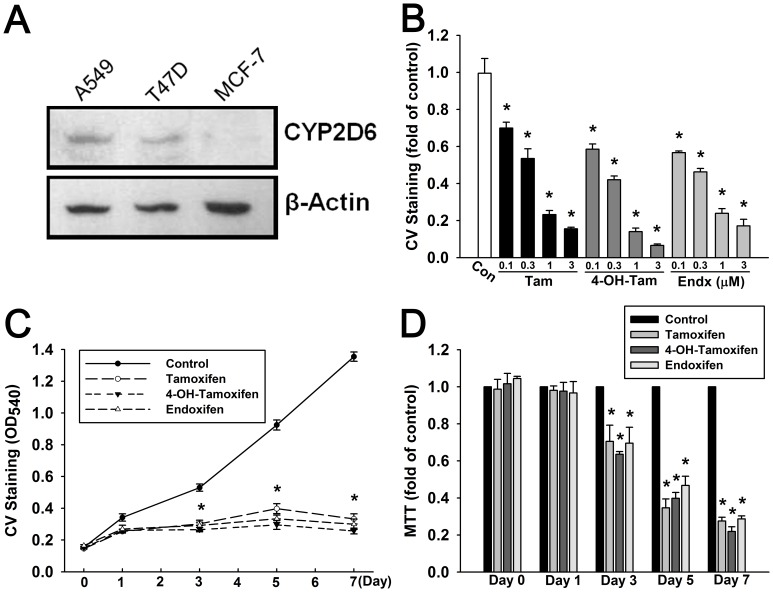
T47D human breast cancer cells exhibit tamoxifen-induced cytotoxic effect dose- and time-dependently. (**A**) T47D human breast cancer cells but not MCF-7 cells expressed significant amount of CYP2D6 protein. A549 human lung cancer was loaded as positive control which shows prominent protein expression. (**B**) Cells were treated with tamoxifen, 4-OH-tamoxifen, and endoxifen (0.1–3 µM) for 7 days. Cell viability of T47D cells was examined by crystal violet (CV) staining (**C, D**) Tamoxifen (1 µM), 4-OH-tamoxifen (1 µM), and endoxifen (1 µM) markedly inhibited cell viability from Day3 to Day7 in T47D cells, measured by both crystal violet (CV) staining and MTT assay. Graphs show mean ± S.E.M. of at least three independent experiments. *, *p*<0.05 to control group; t-test. Tam, tamoxifen; 4-OHTam, 4-hydroxy-tamoxifen; Endx, endoxifen.

In order to validate that T47D human breast cancer is suitable for this designed research, we firstly examined several human breast cancer cell lines whether they express CYP2D6 enzyme. A549 human lung cancer cell line was used as positive control which shows prominent CYP2D6 protein expression. As shown in Figure 1A, T47D human breast cancer cells showed significant amount of CYP2D6 protein, while MCF-7 cells did not express detectable protein amount. Hence, we used T47D cell line as the main material in the following experiments. We further confirmed that tamoxifen, as well as 4-OH-tamoxifen and endoxifen, exerted cytotoxic effect dose-dependently in a low-dosage range (0.1–3 µM) (Figure 1B), suggesting that T47D cells could metabolize tamoxifen (prodrug) to active metabolites. As a cytostatic agent, tamoxifen revealed its cytotoxicity after 3 days (Figure 1C and D). In accordance with tamoxifen, 4-OH-tamoxifen and endoxifen also showed similar effects from 3 days to 7 days. At Day 7, T47D cell number was only 0.28-fold to control upon 1 µM tamoxifen treatment, and 0.22-fold and 0.29-fold to control upon 1 µM 4-OH-tamoxifen or 1 µM endoxifen treatment, respectively.

**Figure 2:**
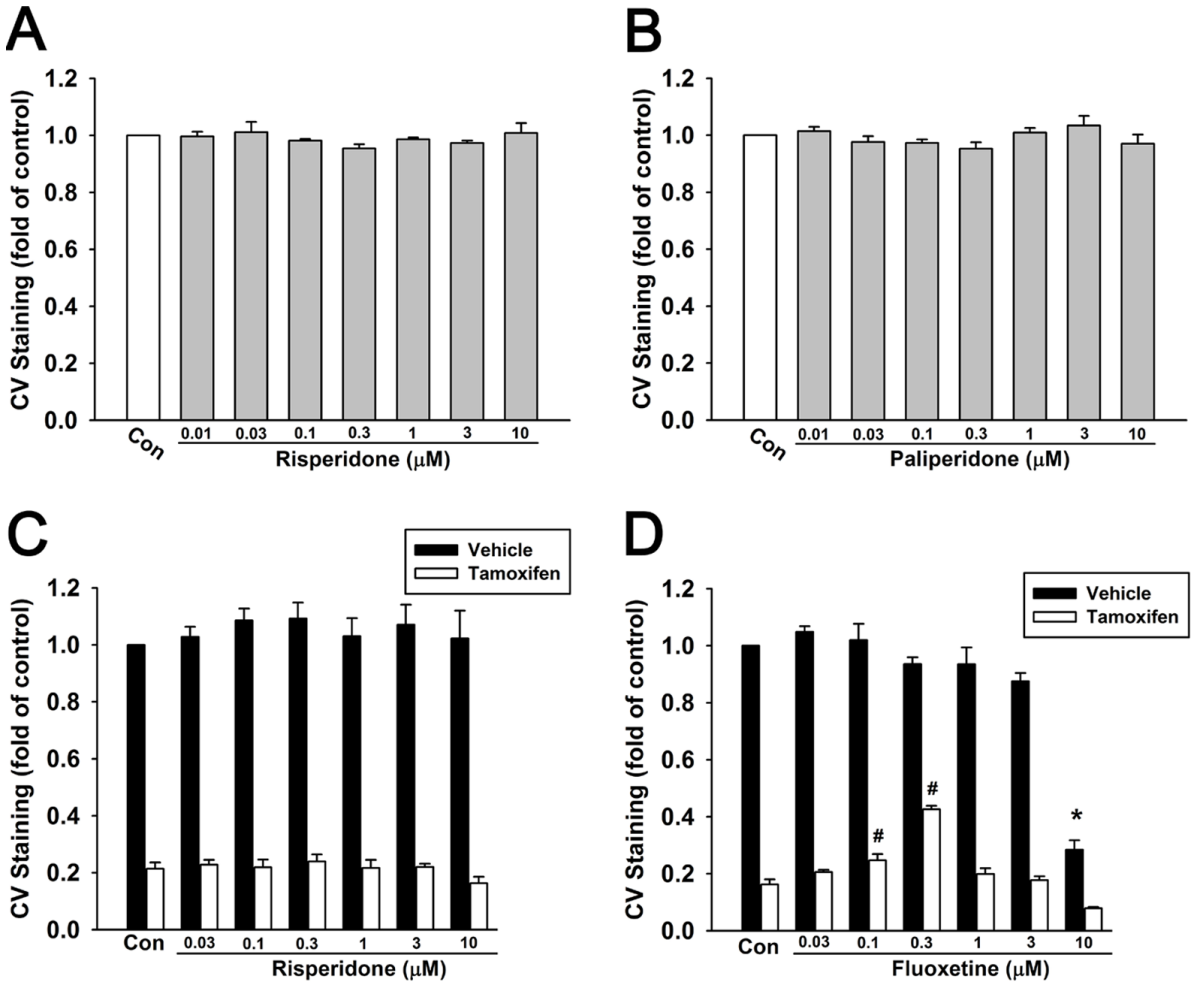
Risperidone shows no interference in tamoxifen-induced cytotoxic effect in T47D cells. Cells were treated with risperidone (**A**) or its main metabolite paliperidone (0.01–10 µM) (**B**) for 7 days. Cells were treated with control vehicle (closed bar) or 1 µM tamoxifen (open bar) with or without risperidone (**C**) or fluoxetine (**D**) for 7 days. Cell viability of T47D cells was examined by crystal violet (CV) staining. Graphs show mean ± S.E.M. of at least three independent experiments. *, *p*<0.05 to control vehicle group; #, *p*<0.05 to control tamoxifen alone group; t-test.

To investigate the possibility of combination of tamoxifen and risperidone, we then demonstrated whether risperidone counteracts the effects of tamoxifen. Firstly, we found that risperidone and its major active metabolite paliperidone did not affect cell viability from 0.01 µM to 10 µM in T47D cells (Figure 2A and B). In combination with risperidone, tamoxifen (1 µM)-induced cytotoxicity in T47D cells was not affected in the dosage range of 0.03–10 µM risperidone (Figure 2C). As a known CYP2D6 inhibitor, fluoxetine was used as a positive control to antagonize tamoxifen-induced cytotoxicity. Although high dose fluoxetine (10 µM) alone induced significant cell death, 0.1 µM or 0.3 µM fluoxetine in combination with 1 µM tamoxifen resulted in reduced cell death compared with tamoxifen alone (Figure 2D). These results suggested that fluoxetine antagonized tamoxifen-induced cytotoxicity and resulted in reduced amount of cell death, while risperidone did not exert affection in combination with tamoxifen.

### Cell cycle arrest induced by tamoxifen is not interfered by risperidone in breast cancer cells

**Figure 3:**
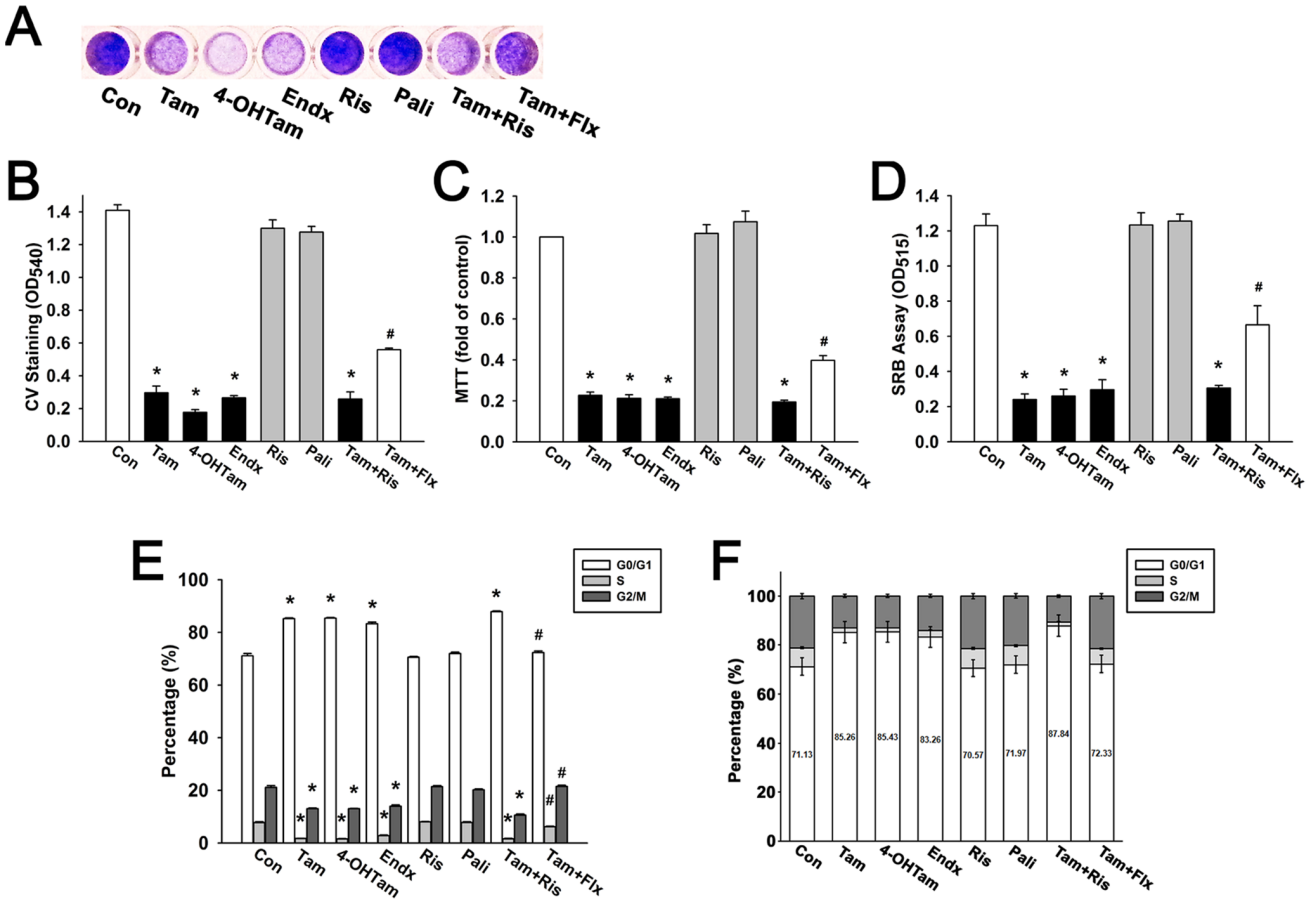
Tamoxifen-induced cell cycle arrest in G0/G1 phase is not interfered by risperidone in T47D cells. Cells were treated with tamoxifen (1 µM), 4-OH-tamoxifen (1 µM), endoxifen (1 µM), risperidone (3 µM), paliperidone (3 µM), tamoxifen with 3 µM risperidone, or tamoxifen with 0.3 µM fluoxetine for 7 days. Cell viability was examined by crystal violet staining (**B**), MTT assay (**C**), and SRB assay (**D**). Representative crystal violet staining was shown as (**A**) and quantified by spectrophotometry (**B**). (**E**, **F**) Tamoxifen-induced cytostasis analyzed by flow cytometry showed that cell cycle was arrested at G0/G1 phase, since the percentage of cells at G0/G1 phase was markedly increased, and percentage of cells at S and G2/M phase were decreased respectively by tamoxifen treatment for 2 days. Graphs show mean ± S.E.M. of at least three independent experiments. *, *p*<0.05 to control group; #, *p*<0.05 to tamoxifen-treated group; t-test. Tam, tamoxifen; 4-OHTam, 4-hydroxy-tamoxifen; Endx, endoxifen; Ris, risperidone; Pali, paliperidone; Flx, fluoxetine.

Attempting to confirm that risperidone does not counteract the effects of tamoxifen, we examined the efficacy of tamoxifen into different aspects, and observed whether risperidone reveals any influence. As shown in Figure 3B, the absolute OD_540_ value of CV staining of control T47D cells was 1.41±0.03 in a 96-well plate after culturing 7 days. Tamoxifen (1 µM), 4-OH-Tam (1 µM), and endoxifen (1 µM) exerted growth inhibitory effect by reducing the OD values to 0.30±0.04, 0.18±0.02, and 0.27±0.01 on Day 7, respectively. In combination with 3 µM risperidone, tamoxifen-induced growth inhibitory effect was maintained around 0.26±0.04. However, in combination with 0.3 µM fluoxetine, tamoxifen-induced growth inhibitory effect was affected, showing a value of 0.56±0.01. A representative crystal violet staining image was shown in Figure 3A. MTT assay also demonstrated accordant results that in combination with risperidone, tamoxifen caused cell viability down to 0.19-fold to control, while fluoxetine antagonized the effect of tamoxifen to only 0.40-fold to control (Figure 3C). Moreover, SRB assay exhibited similar outcomes that the OD_515_ value of tamoxifen plus risperidone was not markedly different from the OD_515_ value of tamoxifen alone (0.31±0.02 and 0.24±0.03, respectively), while the OD_515_ value of tamoxifen plus fluoxetine significantly raised to 0.67±0.11 (Figure 3D). In other words, risperidone showed no noticeable interference with tamoxifen in T47D cells, while fluoxetine antagonized tamoxifen-induced cytotoxicity and resulted in reduced amount of cancer cell death.

**Figure 4:**
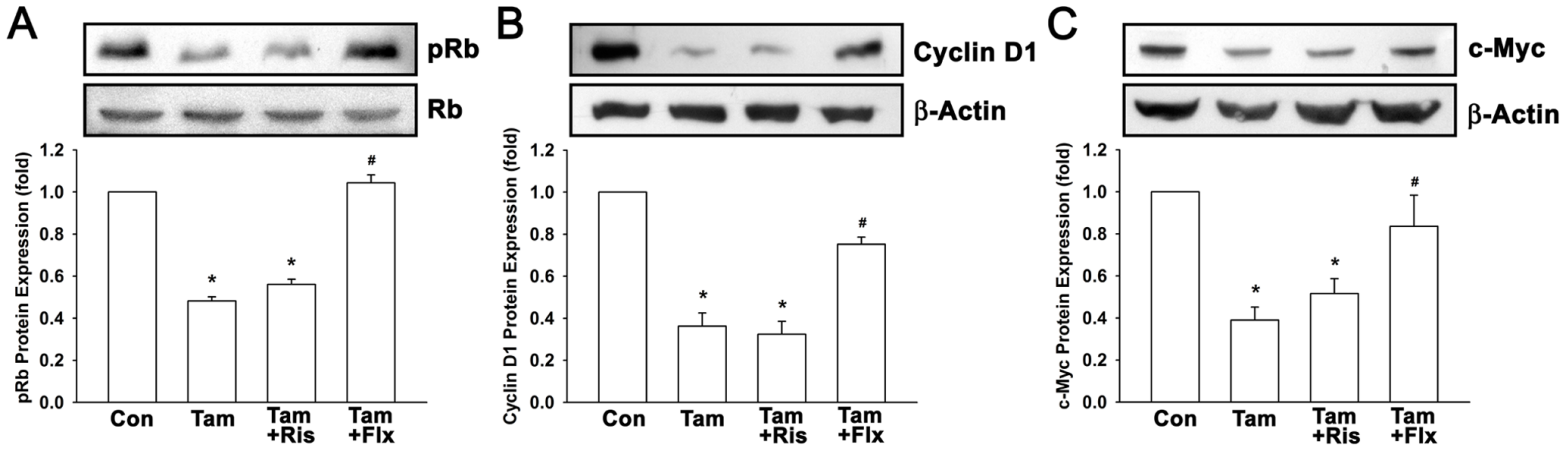
Tamoxifen down-regulates cell cycle regulators pRb, cyclin D1 and oncoprotein c-Myc without disturbing by risperidone. Cells were treated with 1 µM tamoxifen with or without 3 µM risperidone or 0.3 µM fluoxetine for 48 hours. Protein expression of cell cycle regulators pRb (A) and cyclin D1 (B) and oncoprotein c-Myc (C) were measured by Western blotting. Graphs show mean ± SEM of three or more independent experiments. *, *p*<0.05 to control group; #, *p*<0.05 to tamoxifen-treated group; t-test. Tam, tamoxifen; 4-OHTam, 4-hydroxy-tamoxifen; Endx, endoxifen; Ris, risperidone; Pali, paliperidone; Flx, fluoxetine.

In order to determine the effects of combination treatment on cell cycle progression, T47D cells were treated with tamoxifen and risperidone or fluoxetine for 48 hours, and then flow cytometry was performed on propidium iodide stained cells. As shown in Figure 3E and F, the percentage of G_0_/G_1_, S, and G_2_/M phase cells in control group were 71.13%±0.84, 7.74%±0.33, and 21.13%±0.67, respectively. In tamoxifen (1 µM) treated group, the percentage of G_0_/G_1_ phase cells was significantly elevated to 85.26%±0.27, and the percentage of S and G_2_/M phase cells were markedly decreased to 1.72%±0.04 and 13.02%±0.27, respectively. In combination with risperidone (3 µM), tamoxifen-induced cytostatic effect was maintained around the same level as tamoxifen alone group (87.84%±0.29 in G_0_/G_1_ phase). However, in combination with fluoxetine (0.3 µM), the percentage of G_0_/G_1_ phase cells was dropped to 72.33%±0.62, and the percentage of S and G_2_/M phase cells went up to 6.19%±0.17 and 21.47%±0.50, respectively. These results suggested that fluoxetine antagonized tamoxifen-induced cytostasis while risperidone showed no marked influence. Protein expression of cell cycle regulators cyclin D1 and retinoblastoma protein (Rb) and oncoprotein c-Myc were further examined. Cyclin D1 binds to cyclin-dependent kinase (CDK) which subsequently phosphorylates Rb, and the cells are progressed through G_1_ to the S phase of the cell cycle [43], [44], [45]. Oncoprotein c-Myc is also a positive regulator of G_1_-specific CDK [4], [46], [47], implicated as a direct regulator of the cell cycle machinery. As shown in Figure 4A, treatment of T47D cells with tamoxifen (1 µM) in the absence or presence of risperidone (3 µM) decreased pRb protein expression to 0.48-fold and 0.56-fold to control, respectively, and tamoxifen-fluoxetine combination treatment reversed tamoxifen-induced effect to 1.04-fold to control. Similarly, tamoxifen down-regulated cyclin D1 and c-Myc to 0.36-fold and 0.39-fold to control, respectively, while risperidone exerted no significant interference (Figure 4B and C). Nevertheless, tamoxifen-fluoxetine combination treatment caused tamoxifen-induced down-regulation of cyclin D1 and c-Myc went back up to 0.75-fold and 0.84-fold to control, respectively. These results suggested that tamoxifen-induced down-regulation of pRb, cyclin D1, and c-Myc protein expression were markedly affected by fluoxetine, however, risperidone did not affect tamoxifen-induced effects.

### Cell apoptosis resulted from tamoxifen is not influenced by risperidone in breast cancer cells

**Figure 5:**
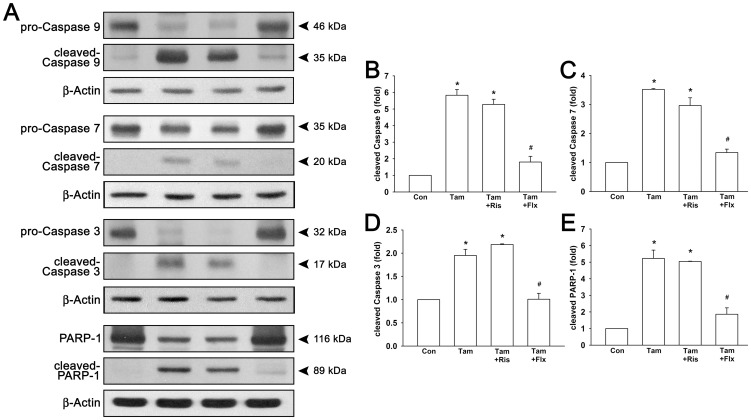
Risperidone has no influence on tamoxifen-induced cleavage of caspases and PARP-1 in T47D cells. Cells were treated with 1 µM tamoxifen with or without 3 µM risperidone or 0.3 µM fluoxetine for 72 hours. Representative protein blotting images are shown in (**A**). Treatment of tamoxifen with or without risperidone resulted in increased protein expression of cleaved caspase 9 (**B**), caspase 7 (**C**), caspase 3 (**D**), and PARP-1 (**E**). Graphs show mean ± SEM of three or more independent experiments. *, *p*<0.05 to control group; #, *p*<0.05 to tamoxifen-treated group; t-test. Tam, tamoxifen; Ris, risperidone; Flx, fluoxetine.

Cell apoptosis may be a consequence of cell cycle arrest when cells do not tolerate cytostatic condition. Based on accumulating evidence suggesting activation of caspase-dependent apoptosis by tamoxifen [5], [22], [23], we examined the effects on caspases cleavage and expression by treatment of tamoxifen with or without risperidone. As shown in Figure 5, tamoxifen treatment induced pronounced cleavage of caspase 9, caspase 7, and caspase 3 (5.83±0.35-fold, 3.52±0.03-fold, and 1.95±0.13-fold to control, respectively). In combination with risperidone, tamoxifen-induced cleavage of caspases were maintained around the same levels as tamoxifen alone group. Nevertheless, in combination with fluoxetine, cleavage of caspase 9, caspase 7, and caspase 3 were down to 1.80±0.35-fold, 1.34±0.12-fold, and 1.01±0.13-fold, respectively. Furthermore, PARP-1, the known substrate of caspase 7 and caspase 3 was also detected. Similarly, both tamoxifen alone group and tamoxifen-risperidone combination group resulted in elevated levels of cleaved PARP-1 to 5.23±0.5-fold and 5.04±0.02-fold to control. However, combination of tamoxifen and fluoxetine increased cleaved PARP-1 to only 1.86±0.39-fold.

**Figure 6:**
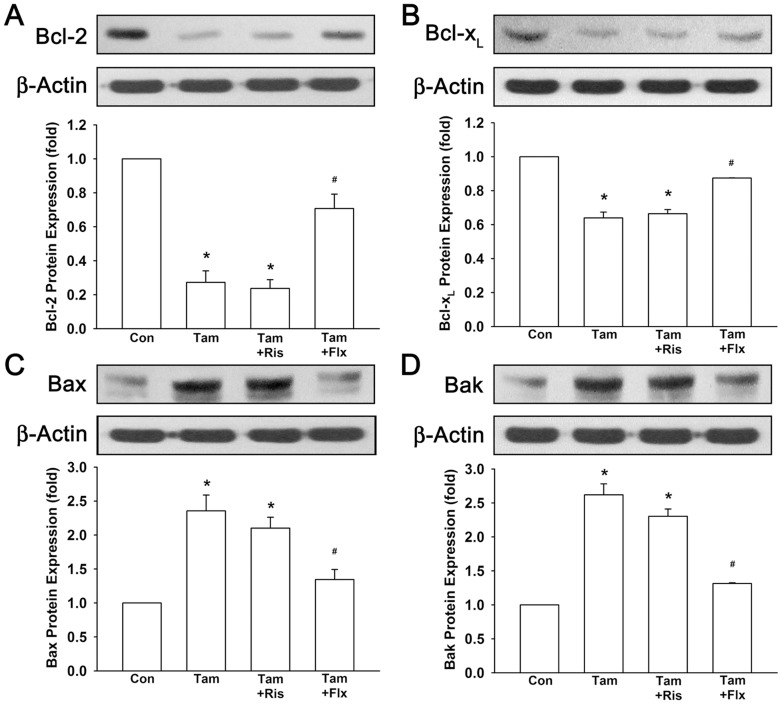
Regulation of anti-apoptotic and pro-apoptotic protein expression caused by tamoxifen are not affected by risperidone. Cells were treated with 1 µM tamoxifen with or without 3 µM risperidone or 0.3 µM fluoxetine for 72 hours. Treatment of tamoxifen with or without risperidone resulted in decreased protein expression of Bcl-2 (A) and Bcl-x_L_ (B). Protein expression of Bax (C) and Bak (D) were increased by tamoxifen with or without risperidone. Graphs show mean ± SEM of three or more independent experiments. *, *p*<0.05 to control group; #, *p*<0.05 to tamoxifen-treated group; t-test. Tam, tamoxifen; Ris, risperidone; Flx, fluoxetine.

In addition, expression of anti-apoptotic and pro-apoptotic proteins were also investigated. As shown in Figure 6A and B, protein expression of anti-apoptotic Bcl-2 and Bcl-x_L_ were down-regulated to 0.27±0.07-fold and 0.64±0.03-fold to control by tamoxifen. In combination with risperidone, Bcl-2 and Bcl-x_L_ were also decreased to 0.24±0.05-fold and 0.66±0.22-fold by tamoxifen. Protein expression of pro-apoptotic Bax and Bak were up-regulated to 2.35±0.23-fold and 2.62±0.16-fold to control by tamoxifen (Figure 6C and D). In combination with risperidone, Bax and Bak were also increased to 2.10±0.16-fold and 2.30±0.11-fold by tamoxifen. In all cases, combination treatment of fluoxetine and tamoxifen abrogated the effects induced by tamoxifen alone. These results indicated that while fluoxetine disturbed the effects of tamoxifen, combination of risperidone and tamoxifen exerted similar tamoxifen efficacy without significant interference.

### Stress responses of endoplasmic reticulum caused by tamoxifen is not affected by risperidone in breast cancer cells

**Figure 7:**
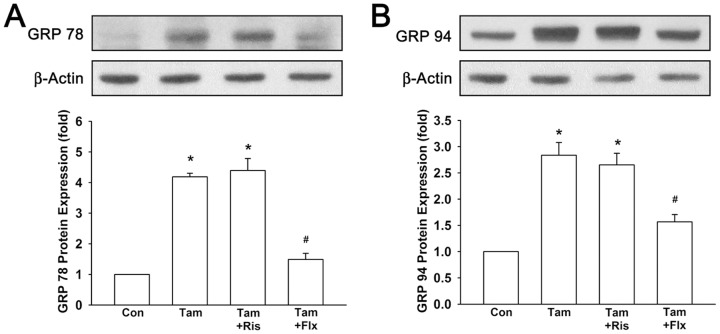
Tamoxifen-induced stress responses of endoplasmic reticulum are not interfered by risperidone. Tamoxifen-induced protein expression of GRP 78 (**A**) and GRP 94 (**B**) are not affected by risperidone. Graphs show mean ± SEM of three or more independent experiments. *, *p*<0.05 to control group; #, *p*<0.05 to tamoxifen-treated group; t-test. Tam, tamoxifen; Ris, risperidone; Flx, fluoxetine.

There is increasing evidence that stress of endoplasmic reticulum plays an important role in the regulation of apoptosis. It has been reported that endoplasmic reticulum stress triggers several signaling pathways such as glucose-regulated protein (GRP) 78 and GRP 94 [48]. GRPs are the most abundant glycoproteins in the endoplasmic reticulum and play critical roles in endoplasmic reticulum regulation [49]. As shown in Figure 7, tamoxifen induced GRP 78 to 4.19±0.12-fold to control and GRP 94 to 2.84±0.24-fold to control. In combination of risperidone and tamoxifen, GRP 78 was increased to 4.39±0.39-fold to control and GRP 94 was increased to 2.65±0.22-fold to control. However, in combination of fluoxetine and tamoxifen, tamoxifen-induced elevated GRP 78 and GRP 94 were significantly reversed to a lower extant (1.49±0.2-fold and 1.57±0.14-fold to control, respectively). These data suggested that risperidone does not have notable influence on effects of tamoxifen.

### Reduction of tumor volume exerted by tamoxifen is not interfered by risperidone

**Figure 8:**
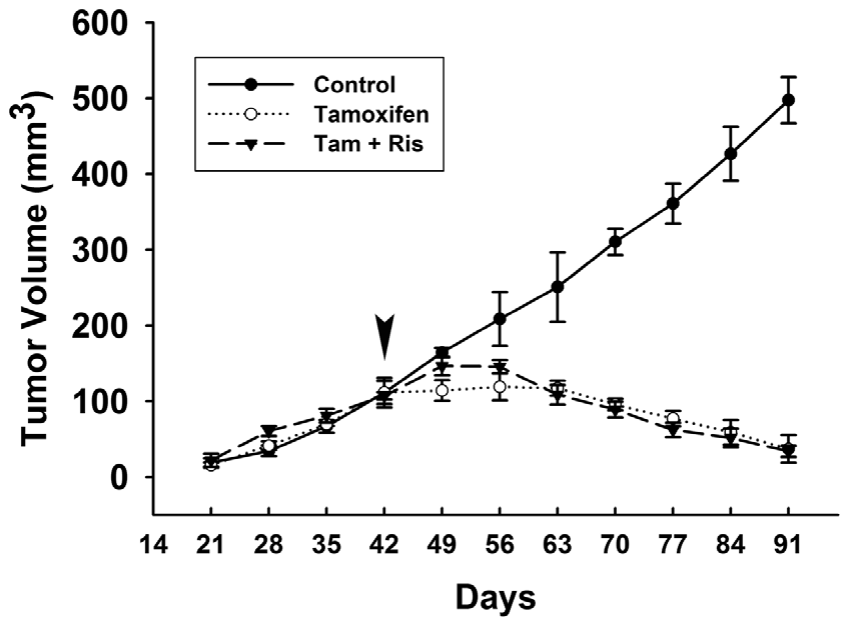
Decrease of tumor volume by tamoxifen is not influenced by combination treatment of risperidone. In T47D-inoculated female nude mice, tumor masses had been recorded since day 21, and the individual or combination regimens of each drug [25 µg tamoxifen (Tam) per mouse or/and 2.5 µg risperidone (Ris) per mouse] were started at Day42 with intraperitoneal injection every 2 days. Tumor volume (tumor volume  =  length×width^2^×0.5) was recorded once a week until Day 91. Graph shows mean ± SEM of four mice in each group.

In addition to above mentioned *in vitro* experiments based on T47D breast cancer cells, we further performed *in vivo* study to confirm the accordance in animals. We inoculated T47D breast cancer cells into the mammary gland of female nude mice, and palpable tumors were observed since Day 21. As shown in Figure 8, tumor volume had increasingly enlarged in all groups. Clinically, the initial dose of tamoxifen has been established to be 20 mg daily (approximately 0.33 mg/kg) in breast cancer patients, and high-dose tamoxifen over 100 mg daily may be given in advanced breast cancer but not administered long-term [50]. In our study, we had titrated the effective dose of tamoxifen from 0.33 mg/kg to 1 mg/kg (3 times of initial dosage in patients, approximately 25 µg was given per mouse) (data not shown). On the other hand, the dosage of risperidone for hot flushes in clinical trial [32] was 2 mg (0.033 mg/kg) daily. Hence, we applied 3 times of the risperidone dosage (0.1 mg/kg, approximately 2.5 µg per mouse) in animal model. From Day 42, regimens of tamoxifen alone or combination of tamoxifen and risperidone were given every other day until mice were sacrificed. Both regimens resulted in slower increased of tumor size from Day 42, and shrinkage of tumor volume was observed since Day 56. This data suggested that combination of risperidone and tamoxifen does not affect the efficacy of tamoxifen.

## Discussion

Tamoxifen is the most commonly used SERM for the treatment of both early and advanced ER-positive breast cancer [8], [9], [10], [11]. Tamoxifen exerts its cytotoxic effect primarily through cytostatic action which is associated with the accumulation of cells in the G_0_/G_1_ phase of the cell cycle [20]. Moreover, cytostasis, induced by cell cycle arrest, is a condition that is poorly tolerated by any cell, hence the apoptotic activity of these primarily cytostatic agents [21]. It has been reported that tamoxifen-induced apoptosis involves cleavage of caspase 9, caspase 7, caspase 3, and poly-ADP-ribose polymerase (PARP) [5], [22], [23]. Anti-apoptotic protein Bcl-2 and pro-apoptotic protein Bax are also important effectors in the regulation of tamoxifen-induced cell death [5], [24]. In addition, endoplasmic reticulum stress has also been reported to play a crucial role in the regulation of apoptosis [38], [51]. There is increasing evidence indicates that stress responses of endoplasmic reticulum trigger several signaling pathways, such as glucose-regulated protein (GRP) 78 and GRP 94 [49]. Pro-apoptotic Bcl-2 family members like Bax, has also been shown to be involved in endoplasmic reticulum stress-induced cell death [52], [53]. However, this mechanism is seldom discussed in tamoxifen-treated breast cancer cells.

Hot flushes, the most prevalent menopause-related vasomotor symptoms, is a major problem for breast cancer patients [54]. Studies reported that approximately 65% of women after treatment for breast cancer experiencing hot flushes, and the incidence is even higher among tamoxifen users (72%) [54], [55], [56].The physiology of hot flushes is associated with reduced hormone levels that affect the thermoregulatory system in the body and result in the sensations of heat [57]. The mechanism on the genesis of hot flushes is a strong reduced estrogen levels leads to a significant reduction of the blood serotonin level and consequently to an up-regulation of 5-HT_2A_ receptors. The hypothalamic temperature set point is disturbed by extra released 5-HT and activation of 5-HT_2A_ receptors, and autonomic reactions to cool down the body cause increased skin temperature [58], [59]. This symptom is described as episodic flushing, sweating, and are often accompanied by heart palpitations and feeling of anxiety. Hot flushes that occur at night can also interrupt sleep [60]. These symptoms not only can greatly decrease quality of life, but they may lead to discontinuation of cancer therapies such as tamoxifen and aromatase inhibitors [13], [14], [61]. Hormone replacement therapy (HRT) that used for menopause hot flushes is a controversial option for breast cancer patients because of a relationship among estrogen and/or progesterone and breast cancer recurrence and mortality [62], [63]. Among nonhormonal treatment alternatives, SSRIs antidepressants are one of the most commonly prescribed options for hot flushes in breast cancer patients [64], [65], [66]. Unfortunately, many SSRIs such as fluoxetine and paroxetine are known to strongly inhibit cytochrome P450 2D6 (CYP2D6) which is an essential isoenzyme for tamoxifen metabolism. Tamoxifen, as a prodrug, is metabolized in the liver mainly by CYP2D6 isoenzyme to active metabolites [15]. The inhibition of CYP2D6 decreases tamoxifen metabolism and adversely affects the efficacy of breast cancer treatment [16], [17]. Evidence shows that co-administration of CYP2D6 inhibitor like fluoxetine or paroxetine decreases the plasma concentration of tamoxifen metabolites [18], [19]. The women taking tamoxifen alone had an overall 7.5% recurrence rate while the women taking tamoxifen with SSRIs (fluoxetine, paroxetine, or sertraline) had a 13.9% recurrence rate [67]. It has also been reported that taking paroxetine during tamoxifen treatment is associated with an increased breast cancer mortality [68]. Another retrospective patient cohort also showed that decreased CYP2D6 metabolism results in increased rates of breast cancer recurrence and decreased relapse-free survival time [16], [69]. These strongly implicate the SSRI antidepressants as having a negative drug interaction with tamoxifen. Hence, searching for an ideal drug for relieving hot flushes without disturbing the efficacy of tamoxifen is greatly important in tamoxifen-treated breast cancer patients.

Risperidone, a 5-HT_2A_ antagonist, has been tested for treating hot flushes in hysterectomy, menopause, and perimenopause women whose hot flushes were caused by over activation of 5-HT_2A_ receptors [32]. Unlike other commonly used SSRIs for these symptoms, risperidone does not inhibit CYP2D6 enzyme activity which is essential for tamoxifen metabolism [25], [33]. Wu demonstrated that risperidone is effective for treating hot flushes with a relatively low dosage [32]. In a clinical study, risperidone was given at a dose of 2 mg per day to menopause woman and the patient reported that the occurrence of hot flushes reduced markedly two days after risperidone treatment and was completely eliminated by day 7. To assess the relationship between risperidone therapy and the resolution of hot flushes, risperidone was tapered off over 2 days, and the patient experienced hot flushes after risperidone discontinued [70]. In a perimenopausal case, risperidone treatment was given at 1 mg per night, and the frequency and intensity of patient's hot flushes were significantly reduced three days after risperidone therapy. Three months later, the dosage of risperidone was decreased to 0.25 mg per day, and the patient's hot flushes were still markedly eliminated. In other patients with hysterectomy or hormone replacement therapy, risperidone also can significantly relieve patient's hot flushes in 2 to 3 days without apparent side effects, and the patent has been issued by the United States in 2010 [32]. Although risperidone has been reported to induce hyperprolactinemia [71], [72], it has been reported that the risk for breast cancer is not evidently increased in women with hyperprolactinemia [73].

As previous evidence has shown that risperidone is effective in treating hot flushes, we hypothesized that risperidone is a potential candidate to combine with tamoxifen instead of SSRIs in breast cancer patients. Hence, we investigated whether risperidone interferes with the effects of tamoxifen. Throughout this study, we have demonstrated that tamoxifen-induced cytotoxic effect was not interfered by combination treatment of risperidone in T47D breast cancer cells (Figure 3B-D). However, fluoxetine, as a positive control, antagonized tamoxifen-induced cell death possibly due to inhibition of CYP2D6 hence reduced tamoxifen efficacy. Moreover, tamoxifen-induced cell cycle arrest in G0/G1 phase was not affected by risperidone treatment (Figure 3E and F). In addition, tamoxifen-induced down-regulation of cell cycle regulators pRb, cyclin D1, and c-Myc were also not affected by the presence of risperidone (Figure 4). In the meanwhile, fluoxetine markedly intervened tamoxifen-induced cell cycle arrest and regulation of pRb, cyclin D1 and c-Myc expression. Furthermore, tamoxifen induced several apoptotic signaling, such as activation and cleavage of caspase 9, caspase 7, caspase 3, and PARP-1 (Figure 5). Anti-apoptotic Bcl-2 and Bcl-x_L_ were observed to be down-regulated by tamoxifen, and pro-apoptotic Bax and Bak were up-regulated by tamoxifen treatment (Figure 6). These apoptosis signalings induced by tamoxifen were not significantly disturbed by the appearance of risperidone in combination with tamoxifen, but markedly abrogated by the addition of fluoxetine. Besides, we also examined the expression of endoplasmic reticulum stress-associated GRP for the first time. Notably, we found that protein expressions of GRP 78 and GRP 94 were pronounced increased by tamoxifen (Figure 7). In combination of tamoxifen and risperidone, GRP 78 and GRP 94 were elevated to similar levels compared to tamoxifen alone group without noticeable influence, while fluoxetine exhibited remarkable disturbance. Finally, we demonstrated tumor xenograft study to determine whether the efficacy of tamoxifen treatment is interfered by risperidone in female nude mice (Figure 8). From our results, both individual (tamoxifen alone) and combination regimens (tamoxifen plus risperidone) reduced growth rate of breast cancer, and shrinkage of tumor volume was observed ultimately. This data suggested that combination of risperidone and tamoxifen does not affect the efficacy of tamoxifen in animal model. Although further animal studies may be needed for confirming the efficacy of risperidone for hot flushes in combination with tamoxifen, this is the first report suggested that risperidone is a potential candidate for treating tamoxifen-induced hot flushes without reducing tamoxifen efficacy against breast cancer.
